# Supplementary material for: The importance of organisational culture for health system resilience: A qualitative analysis of factors that supported health care workers in Germany during the COVID-19 pandemic
Source: PLoS One. 2025 Jan 14;20(1):e0317231. doi: 10.1371/journal.pone.0317231 (PMC11731743; doi:10.1371/journal.pone.0317231)
Supplement: S1 Table — (DOCX) [file pone.0317231.s001.docx]

Table 1: Consolidated criteria for reporting qualitative studies (COREQ): 32-item checklist for the paper “The importance of organisational culture for health system resilience: A qualitative analysis of factors that supported health care workers in Germany during the COVID-19 pandemic”

| **No.** | **Item** | **Guide questions + answers** |
| --- | --- | --- |
| **Domain 1: Research team and reflexivity** | | |
| Personal Characteristics | | |
| 1. | Interviewer/facilitator | *Which author/s conducted the interviews and focus group discussions?*  Heide Weishaar, Rike Böttcher, Rene Umlauf, Barbara Buchberger |
| 2. | Credentials | *What were the researcher's credentials? E.g. PhD, MD*  Three researchers held a PhD, and one researcher held a Bachelor-degree. |
| 3. | Occupation | *What was their occupation at the time of the study?*  Heide Weishaar: Deputy Head of ZIG2 and Senior Researcher, ZIG2-RKI; Rike Böttcher: Intern, ZIG2-RKI; Rene Umlauf: Senior Researcher, ZIG2-RKI; Barbara Buchberger: Senior Researcher, ZIG2-RKI. |
| 4. | Gender | *Was the researcher male or female?*  Heide Weishaar (F), Rike Böttcher (F), Rene Umlauf (M), Barbara Buchberger (F) |
| 5. | Experience and training | *What experience or training did the researcher have?*  All researchers were trained in data collection and in handling the data collection tools prior to the start of the study. All researchers contributed to the development of the data collection tools. |
| Relationship with participants | | |
| 6. | Relationship established | *Was a relationship established prior to study commencement?*  No relationship was established with the respondents prior to study commencement. |
| 7. | Participant knowledge of the interviewer | *What did the participants know about the researcher? e.g. personal goals, reasons for doing the research*  All participants were informed about the goals of the research project, the background (e.g. rationale, funding, etc.), and the researcher’s role in the project via a participant information sheet. Beyond this, participants were able to ask questions. |
| 8. | Interviewer characteristics | *What characteristics were reported about the interviewer/facilitator? e.g. Bias, assumptions, reasons and interests in the research topic*  None of the authors have any personal, financial, professional, or intellectual bias or a conflict of interest in this project. Heide Weishaar is trained as a physiotherapist and therefore has a biographical interest in the research topic. |
| **Domain 2: study design** | | |
| Theoretical framework | | |
| 9. | Methodological orientation and theory | *What methodological orientation was stated to underpin the study? e.g. grounded theory, discourse analysis, ethnography, phenomenology, content analysis*  The qualitative data was analysed using content analysis. |
| Participant selection | | |
| 10. | Sampling | *How were participants selected? e.g. purposive, convenience, consecutive, snowball*  HCWs were recruited between 1 February and 30 November 2022 through a quantitative survey on stress and coping among HCWs during the COVID-19 pandemic which was conducted as part of the overarching study. Survey participants were recruited via the health facilities in which they worked or via professional networks and organisations. HCWs who completed the survey and expressed an interest to participate in the qualitative arm of the study were contacted individually via email.  Key informants were sampled through an online search where potential interviewees were identified from media and other reports. Consecutively, individuals who had commented on the situation of HCWs during the pandemic were listed as potential participants. Purposive sampling was then undertaken to ensure a diverse selection of key informants from various regions, areas of work, genders, and roles. |
| 11. | Method of approach | *How were participants approached? e.g. face-to-face, telephone, mail, email*  Participants were approached via e-mail. |
| 12. | Sample size | *How many participants were in the study?*  We conducted 10 FGDs with 43 HCWs, individual interviews with 17 HCWs, and 18 individual interviews with key informants. |
| 13. | Non-participation | *How many people refused to participate or dropped out? Reasons?*  A small number of individuals who were asked to participate in a key informant interview refused to participate in the study due to time constraints. For each of these, we made efforts to recruit a key informant with similar characteristics (e.g. type of organization affiliated with, seniority level, role with regard to HCWs during the pandemic, etc.). |
| Setting | | |
| 14. | Setting of data collection | *Where was the data collected? e.g. home, clinic, workplace*  Interviews with key informants were conducted online via videoconference or face-to-face in their workplace, depending on preference of the interviewee. All HCWs’ interviews and FGD were conducted online. |
| 15. | Presence of non-participants | *Was anyone else present besides the participants and researchers?*  No. |
| 16. | Description of sample | *What are the important characteristics of the sample? e.g. demographic data, date*  Key informants included political decision-makers, health facility managers, representatives of professional associations, representatives of health care providers, academics, and other individuals with decision-making functions. |
| Data collection | | |
| 17. | Interview guide | *Were questions, prompts, guides provided by the authors? Was it pilot tested?*  The interview guide contained key questions as well as prompts. It was pilot tested among the research team and three health care workers known to members of the research team. |
| 18. | Repeat interviews | *Were repeat interviews carried out? If yes, how many?*  No repeat interviews were carried out. |
| 19. | Audio/visual recordings | *Did the research use audio or visual recording to collect the data?*  All interviews were audio recorded. |
| 20. | Field notes | *Were field notes made during and/or after the interview?*  Field notes were taken during all interviews. |
| 21. | Duration | *What was the duration of the interviews or focus group?*  Interviews with HCWs and key informants took on average 55 minutes (min: 37 minutes, max: 71 minutes), whereas FGDs lasted on average 70 minutes (min: 57 minutes, max: 79 minutes). |
| 22. | Data saturation | *Was data saturation discussed?*  The field work was ended when data saturation was recorded by the interview teams. |
| 23. | Transcripts returned | *Were transcripts returned to participants for comment and/or correction?*  No, transcripts were not returned for member checking. |
| Domain 3: analysis and findings | | |
| Data analysis | | |
| 24. | Number of data coders | *How many data coders coded the data?*  A random sample of five interviews was independently coded by three researchers. Coding was discussed, and the codebook was revised thoroughly according to consensus. All transcripts were systematically coded by six members of the study team, applying the revised codebook. One third of all transcripts was double coded to ensure coding was consistent. Consistency checks were performed by a senior researcher. |
| 25. | Description of the coding tree | *Did authors provide a description of the coding tree?*  Yes, the research team developed a comprehensive codebook with descriptions of all codes. |
| 26. | Derivation of themes | *Were themes identified in advance or derived from the data?*  A codebook was developed using pre-defined codes that came from the interview topic guides and in vivo codes that were identified from wording used by respondents. |
| 27. | Software | *What software, if applicable, was used to manage the data?*  NVivo® qualitative data management software version 12. |
| 28. | Participant checking | *Did participants provide feedback on the findings?*  No. |
| Reporting | | |
| 29. | Quotations presented | *Were participant quotations presented to illustrate the themes / findings? Was each quotation identified? e.g. participant number*  Yes. |
| 30. | Data and findings consistent | *Was there consistency between the data presented and the findings?*  Yes. |
| 31. | Clarity of major themes | *Were major themes clearly presented in the findings?*  Yes, all major themes are included in the findings section. |
| 32. | Clarity of minor themes | *Is there a description of diverse cases or discussion of minor themes?*  Yes, diverse cases, differences between responses and minor themes are described in the findings. |
