# Supplementary material for: The importance of organisational culture for health system resilience: A qualitative analysis of factors that supported health care workers in Germany during the COVID-19 pandemic
Source: PLoS One. 2025 Jan 14;20(1):e0317231. doi: 10.1371/journal.pone.0317231 (PMC11731743; doi:10.1371/journal.pone.0317231)
Supplement: Attachment — Submitted filename: Comments to authors.docx [file pone.0317231.s002.docx]

**Comments to authors**

Well-written paper on a very important subject, I have suggestions for the authors to consider:

Introduction

- While defining the role of health workers in the resilience of the health system, it is important to know the rationale for using qualitative methods. Please add some literature on the appropriateness of the selected method of the study.
- Some data on the impact of COVID-19 on the German health system, such as the number of cases, healthcare workers infected, and the challenges faced by health staff, would contribute to a better understanding of the context.

Methods

- Please give a table if possible, with disaggregated demographic details of participants, like age, gender, occupation, years in service, and region, according to data availability and relevance
- A note on the reflexivity of the research team would add value to the paper.
- Please refer to [the COREQ guidelines](https://cdn.elsevier.com/promis_misc/ISSM_COREQ_Checklist.pdf) for reporting qualitative research; though most of the elements in the guidelines are reported in the manuscript, some details, such as member checking, gender balance of the research team, educational qualifications, credentials, etc., can be added.

Results

- The study results elaborate on the enablers that supported the HCWs during the pandemic period. It would be nice to have a little more context in which these factors worked as enablers and, if possible, add barriers faced by the HCWs.
- The study team seems to have considered HCWs as a homogenous cohort. The approach is interesting, considering the study focuses on mental health during the pandemic. I wonder if the stress levels and challenges were similar to all the types and categories of staff, for example, an administrator and clinician may be under severe duress during the period but they may be due to different reasons. Is it possible to add a little more nuance by looking at grouped responses on enablers and barriers across common job categories and see if the themes described still hold same?
- The various initiatives are comprehensively captured, but sometimes, they appear to be local initiatives that stem from innovations and adaptations by individuals and institutions facing a crisis. Is it possible to differentiate between organizational (health system, government)support and individual support mechanisms for better clarity?

Discussion

- While it was wonderful to learn about all the supportive factors that helped the system is it possible to know if at least some of these factors are sustainable as the larger objective is about resilience and sustaining these gains is key.
